# A Narrative Review of Revascularization in Chronic Coronary Syndrome/Disease: Concepts and Misconceptions

**DOI:** 10.3390/jpm14050506

**Published:** 2024-05-10

**Authors:** Beatriz Nogueira-Garcia, Marta Vilela, Catarina Oliveira, Daniel Caldeira, Ana Margarida Martins, Miguel Nobre Menezes

**Affiliations:** 1 Serviço de Cardiologia, Departamento de Coração e Vasos, CHULN Hospital de Santa Maria, 1649-028 Lisbon, Portugal; ana.n.garcia@ulssm.min-saude.pt (B.N.-G.); marta.vilela@ulssm.min-saude.pt (M.V.); catarina.s.oliveira@ulssm.min-saude.pt (C.O.); daniel.caldeira@ulssm.min-saude.pt (D.C.); ana.m.l.martins@ulssm.min-saude.pt (A.M.M.); 2 Centro Cardiovascular da Universidade de Lisboa (CCUL@RISE), Faculdade de Medicina, Universidade de Lisboa, 1049-001 Lisbon, Portugal; 3 Laboratório de Farmacologia Clínica e Terapêutica, Faculdade de Medicina, Universidade de Lisboa, 1049-001 Lisbon, Portugal; 4 Faculdade de Medicina, Centro de Estudos de Medicina Baseada na Evidência (CEMBE), 1649-028 Lisbon, Portugal

**Keywords:** chronic coronary artery disease, chronic coronary artery syndrome, personalized medicine, percutaneous coronary intervention, coronary artery bypass surgery

## Abstract

Ischemic heart disease represents a significant global burden of morbidity and mortality. While revascularization strategies are well defined in acute settings, there are uncertainties regarding chronic coronary artery disease treatment. Recent trials have raised doubts about the necessity of revascularization for “stable”, chronic coronary syndromes or disease, leading to a shift towards a more conservative approach. However, the issue remains far from settled. In this narrative review, we offer a summary of the most pertinent evidence regarding revascularization for chronic coronary disease, while reflecting on less-often-discussed details of major clinical trials. The cumulative evidence available indicates that there can be a prognostic benefit from revascularization in chronic coronary syndrome patients, provided there is significant ischemia, as demonstrated by either imaging or coronary physiology. Trials that have effectively met this criterion consistently demonstrate a reduction in rates of spontaneous myocardial infarction, which holds both prognostic and clinical significance. The prognostic benefit of revascularization in patients with heart failure with reduced ejection fraction remains especially problematic, with a single contemporary trial favouring surgical revascularization. The very recent publication of a trial focused on revascularizing non-flow-limiting “vulnerable” plaques adds further complexity to the field. The ongoing debates surrounding revascularization in chronic coronary syndromes emphasize the importance of personalized strategies. Revascularization, added to the foundational pillar of medical therapy, should be considered, taking into account symptoms, patient preferences, coronary anatomy and physiology, ischemia tests and intra-coronary imaging.

## 1. Introduction

Cardiovascular disease (CVD) is the principal cause of morbidity and mortality worldwide [1,2]. In 2019, ischaemic heart disease (IHD) was the most common manifestation of incident CVD with an estimated 5.8 million new cases in the 57 European Society of Cardiology (ESC) member countries and it accounted for an estimated 45.8 million DALYs [1].

Since it is recognized as a global health problem, efforts have been made to clarify and improve the clinical approach to these patients. Major changes have occurred in the management of chronic coronary syndromes (CCSs), with the development of effective medical prevention therapies and the increasing use of revascularization. However, the best strategy is still a matter of debate, especially regarding a conservative approach alone versus adding interventional/surgical therapy, i.e., revascularization.

According to the 2018 and 2019 ESC guidelines [3,4], myocardial revascularization plays an important role in the management of CCS, along with medical therapy. The two main targets are symptom relief and improvement in prognosis, and most recommendations have a class I indication. However, the results of recent randomized clinical trials (RCT), especially the International Study of Comparative Health Effectiveness with Medical and Invasive Approaches (ISCHEMIA) trial [5], have raised concerns about to whom revascularization should be offered, as well as how and when should it be performed. As a result, a conservative approach is now growing to be commonplace in the field, and indeed the more recent American guidelines on revascularization and/or CCS [6,7] are now more restrictive in their indications, which vary from class IIa to IIb. The topic remains, however, an issue of fierce debate among cardiologists.

Given the lack of consensus, a personalized approach seems more relevant than ever. Hence, in this narrative review paper, we provide not only an overview of the most relevant evidence regarding revascularization for chronic coronary disease/syndrome, but also a personalized perspective on the issue, stemming from the authors’ reflections on less commonly mentioned aspects of major clinical trials, together with clinical experience as both interventionalists and/or clinical cardiologists.

## 2. Why Revascularize CCS Patients?

Coronary artery disease (CAD) is dynamic and can unpredictably lead to a major cardiovascular (CV) event, namely myocardial infarction (MI). Optimal medical therapy is essential and recommended for all patients, as reflected in both American and European guidelines [3,6]. Revascularization may be offered additionally with the intention of improving symptoms, exercise capacity and quality of life, or for improving clinical outcomes by reducing major cardiac events, such as CV-related death and MI [8,9].

Patients with angina symptoms were found to be at a higher risk for CV outcomes than patients without angina or ischemia [8]. Some controversy persists regarding the extent of the benefit from revascularization with either coronary artery bypass graft (CABG) or percutaneous coronary intervention (PCI) as an initial management strategy, as compared with optimal medical therapy alone.

### 2.1. Revascularization—The Prognosis Rationale

The prognostic benefit of revascularization comes primarily from the concept that more severe ischemia is associated with a greater likelihood of events, and that a reduction in ischemia by revascularization could therefore reduce CV events. The foundational trial evidence supporting this concept comes from the coronary artery bypass graft (CABG) trials, where the Coronary Artery Bypass Graft Surgery Trialists Collaboration, published 30 years ago, showed that CABG had a favourable impact on prognosis, including all-cause mortality [10]. Notwithstanding, the effect was heavily dependent on the severity of disease, with more extensive disease, especially involving the proximal left anterior descending, having a strong impact on the likelihood of benefit from revascularization. The largest difference was seen in two major subgroups, left main disease and abnormal left ventricular ejection fraction (LVEF) (<50%), with a very large difference in the former. As a result, to this day, trials assessing prognosis have focused primarily on patients without any of these features, or only abnormal LVEF. To date, no major clinical trial has compared revascularization versus a conservative approach in patients with left main disease.

Another rationale for revascularization would potentially be intervening with vulnerable plaques, since many have argued that non-severely obstructive plaques, with vulnerable characteristics to rupture/erosion (thin capsule, high plaque burden, and highly inflammatory with necrosis), are often the culprit lesions in acute coronary syndromes [11,12,13]. To date, only a major randomized trial has assessed this hypothesis. Its results were very recently published and are discussed further as well [14].

#### 2.1.1. Patients without Left Main Disease and Preserved Left Ventricular Ejection Fraction

The Clinical Outcomes Utilizing Revascularization and Aggressive Drug Evaluation (COURAGE) trial [15] was the first major large, randomized trial to assess the prognostic benefit of revascularization, with PCI only, in patients without left main disease and preserved LVEF. The trial included patients with either one or more proximal epicardial coronary artery lesions with a percentage diameter stenosis ≥ 70% and evidence of myocardial ischemia (which was present in 95% of patients), or a percentage diameter stenosis ≥ 80% and classic angina symptoms (remaining patients included). In this trial, PCI did not reduce the risk of death or MI (primary endpoint), nor any individual secondary endpoint, including spontaneous MI. There were, however, many caveats to the trial, if one tries to extrapolate it to the current reality of PCI.

First, there is the issue of disease severity/extension. The threshold for performing revascularization was coronary stenosis severity as visually estimated (i.e., not measured by quantitative coronary analysis), as is often the case in most revascularization trials. However, it has long been described that this approach is prone to significant inter-operator variability, leading most frequently to lesion overestimation, the so-called “oculo-stenotic reflex” [16,17,18,19,20]. One might, however, assume that because 95% of patients had evidence of myocardial ischemia, this was not really an issue. However, the details of these tests have been published: about 60% of included patients performed myocardial perfusion single-photon emission computed tomographic imaging, of which close to 70% had none or mild ischemia (<3 ischemic segments) and just 30% had moderate to severe (≥3 ischemic segments) ischemia [21]. Among patients undergoing treadmill exercise tests (approximately 40% of the population), only 20% had ST segment depression > 2 mm [22]. Furthermore, when considering the distribution of disease, only approximately 30% of patients had three-vessel disease or proximal left anterior descending artery (LAD) involvement [15]. And lastly, the very fact that CABG was ruled out a priori highlights that very extensive disease was excluded. Hence, one might argue that the COURAGE trial seems to have included mostly patients with relatively mild, non-severe, coronary artery disease.

The second issue concerns the revascularization details, especially when viewed from today’s perspective. Among the 1149 patients in the PCI arm, 46 did not undergo the procedure, the lesion was not crossed in 27 patients, and, in 71 patients, a stent was not implanted. Thus, 143 patients (12.4%) were either not treated or received suboptimal revascularization. A total of 59% of patients received only one stent, even though two- or three-vessel disease was present in 69% of the population. And because the trial was conducted in the early years of drug-eluting stents (DESs), only 31 patients (2.7%) received these. Hence, approximately 15% of the population did not receive optimal PCI, by today’s standards.

Crossover was also an issue. The authors projected a cross-over rate from medical therapy to PCI of 3% for the first 2 years, 2% for the next 2 years, and 1% thereafter [15]. Notwithstanding, 32.6% of patients crossed over during the original 2.5- to 7-year follow-up period, with a median time of 11 months, with a much lower rate of 2.7% per year after the first year [23]. Thus, the cross-over rate was much higher than anticipated and occurred early in the trial, hampering potential differences between treatment strategies. Lastly, both arms lost close to 100 patients to follow-up.

Putting it all together, the COURAGE trial seems to have included a population with mostly non-extensive coronary artery disease and non-severe ischemia, where many patients in the PCI arm received either none or suboptimal revascularization, and a large proportion of the conservative arm received early revascularization. Furthermore, the trial predates the advent of DESs, which are associated with a markedly decreased incidence of stent restenosis, repeated revascularizations, and stent thrombosis, and does not therefore represent today’s standards of PCI at all [24,25,26].

Despite all of this, the COURAGE trial remains a landmark study, as it reflected common clinical practice, especially at the time: performing PCI based solely on angiographic criteria plus symptoms or evidence of (often mild) ischemia on a treadmill or imaging test. This was undoubtedly a brave design and the trial’s name was therefore most fitting and allowed us to understand that optimal medical therapy is very effective in patients with relatively mild coronary artery disease/ischemic extension, where PCI does not seem to offer an increased prognostic value. The compliance achieved during the trial (in both arms) showed high optimal medication use, a very significant reduction in LDL levels, high levels of appropriate diet, exercise improvement and smoking cessation, with the help of dedicated nurses. While this cannot probably be extrapolated to the “real world”, where such optimal conditions are seldom present, it proves how impactful such a dedicated approach could be.

Another influential trial was the Bypass Angioplasty Revascularization Investigation 2 Diabetes (BARI-2D) trial [27], which explored the issue of either CABG or PCI in patients with obstructive CAD (as defined by ≥50% diameter stenosis and a positive ischemia test, or ≥70% diameter stenosis and typical angina) and diabetes. The trial showed that CABG, but not PCI, was associated with a reduction in major adverse cardiovascular events, but not overall mortality. The trial shares several limitations with the COURAGE trial, and the superior impact of CABG was likely not only the result of its superior performance in diabetic patients as demonstrated posteriorly in the Future Revascularization Evaluation in Patients with Diabetes Mellitus: Optimal Management of Multivessel Disease (FREEDOM) trial [28], but also due to the fact that the surgical patients had far more extensive disease, and were therefore more likely to benefit from revascularization.

Somewhat contemporarily to the COURAGE trial, the Deferral Versus Performance of PTCA in Patients Without Documented Ischemia (DEFER) [29] and Fractional Flow Reserve Versus Angiography for Multivessel Evaluation (FAME) [30] trials also brought important insights to the issue of PCI in CCS. Both trials employed ischemia testing as a decision method to proceed with revascularization, by using an invasive coronary physiology (FFR–index) rather than imaging or ECG treadmill tests.

The DEFER trial tested if PCI was beneficial in patients with an FFR > 0.75. There were no differences in either outcomes or symptoms in patients above the 0.75 cut-off, thereby suggesting that PCI above the FFR ischemic threshold is unnecessary.

The FAME trial compared angiography-guided (based on diameter stenosis visual estimation) vs. FFR-guided (based on a FFR value of ≤0.80) PCI. The latter group experienced improved outcomes, with a reduced rate of a composite outcome of death, MI and repeat revascularization, driven primarily by the latter two endpoints, both of which narrowly missed statistical significance on their own. Importantly, PCI was less common in the FFR arm, where more than one-third of lesions had a negative (i.e., >0.80) FFR measurement. Furthermore, 40% of patients in the FAME trial had 70–90% diameter stenosis lesions and 20% had >90% percentage diameter stenosis lesions, by visual assessment. In the COURAGE trial, the mean diameter stenosis using the same method was about 80%. While a direct comparison is not possible, it seems likely that a sizable proportion of patients in the COURAGE trial would have had a negative FFR evaluation, given their seemingly similar angiographic severity.

More recently, angiography-derived FFR with digital software has been developed. One of the most widely studied indexes quantitative flow ratio (QFR) has been shown to correlate strongly with FFR, enabling physiology assessment without engaging in invasive measurements but rather coronary angiography (CAG) images alone. A large randomized clinical trial, the angiographic quantitative flow ratio-guided coronary intervention (FAVOUR III China) [31], used a “FAME-like” approach, testing a QFR-based vs. angiographic-based strategy for deciding on revascularization. The results were, once again, favourable to the physiology arm, with a positive impact on clinical outcomes.

The second major randomized trial that tested whether PCI added to optimal medical therapy could offer a prognostic benefit was the FAME 2 (Fractional Flow Reserve versus Angiography for Multivessel Evaluation 2) trial [32], published a few years after the COURAGE trial. The trial included mostly patients with one-vessel disease (>50%), proximal/mid LAD (close to two-thirds of the population) involvement and normal LVEF (>80% of the population). FFR-guided PCI was associated with a reduction in clinical events (the primary end point was a composite of death, MI and urgent revascularization) compared with a conservative approach. The benefit of PCI was particularly pronounced among patients who had lesions with an FFR < 0.65, where the hazard relative risk reduction for the primary endpoint was 92% (hazard ratio 0.08; 95% confidence interval [CI] 0.03–0.26), a significant *p*-value for interaction (0.010).

The trial was strongly criticized for two major reasons: early termination (due to a very large early difference favouring the FFR arm), as well as the fact that the difference between the two strategies was mainly driven by an increase in the need for urgent revascularization in the medical-therapy group, which is of less importance than “harder” endpoints, such as MI or death. Notwithstanding, there was indeed a significant reduction in MI, maintained through the 5-year follow-up [33], with a relative risk reduction of 33% (hazard ratio 0.66; 95% CI 0.42–1.00). The FAME-2 trial therefore showed, for the very first time in an RCT, that PCI could offer a relevant prognostic benefit in the setting of CCS, in addition to medical therapy, provided there was significant ischemia as demonstrated by FFR, rather than angiographic assessment alone or ischemia on non-invasive testing.

Thus, taken together, the results of the “original” three FFR trials (DEFER, FAME and FAME-2), together with a more recent “digital” approach (angio-derived FFR–QFR), are in agreement and further emphasize the limitations of angiography-based revascularization in patients with CCS. Furthermore, it is likely that the use of intra-coronary imaging for guiding PCI, which has been shown to improve outcomes [34], may further improve the results of PCI in this setting in the future.

The last and largest randomized trial to address the issue of revascularization prognostic impact in CCS was the ISCHEMIA trial [5]. The trial design was somewhat complex. Patients were primarily selected if moderate or severe ischemia was present on non-invasive testing (mostly imaging). Afterwards, Coronary Computed Tomography Angiography (CCTA) was used as a “gatekeeper”, mostly to exclude either patients with left main disease or patients without significant obstructive coronary artery disease. Patients with an LVEF < 35% were also excluded. After these steps, patients were randomized to either an invasive approach (i.e., invasive CAG) or a conservative approach. Revascularization was undertaken with either PCI or CABG, as judged by the local heart team. Angiography percentage diameter stenosis (visual assessment) was used for decisions on revascularization, together with FFR and/or Intravascular Ultrasound (IVUS).

The ISCHEMIA trial was formally negative, with no differences in the primary endpoint (MI, hospitalization for unstable angina, heart failure or resuscitated cardiac arrest) or mortality between arms. Much like the COURAGE trial, there are complex caveats to it, and, in our view, three major misconceptions are often encountered.

The first misconception is that the ISCHEMIA trial was purely a trial of revascularization vs. medical therapy, like the COURAGE or FAME-2 trials. If that was the case, two major prerogatives would be required: the ubiquitous presence of obstructive coronary artery disease from a revascularization perspective (i.e., revascularization would be potentially needed in all patients) and the feasibility of optimal revascularization (i.e., revascularization is feasible with an expected good technical result). As it happens, none of the above were ubiquitous. Indeed, only approximately 80% of the invasive arm received revascularization (76% in the initial months and the remaining 4% later on) because in the 20% that did not, approximately two-thirds had non-significant disease based on invasive CAG and the remainder had disease unamenable to any mode of revascularization. And among those who actually underwent revascularization, 7% of PCI patients did not receive a stent and 8% of CABG patients did not receive an internal mammary artery graft, considered the gold-standard of optimal revascularization. This accounts to approximately 150 (6%) patients. Thus, more than 25% of patients in the invasive arm either did not receive any revascularization or received suboptimal revascularization. On the other hand, nearly a quarter of patients in the conservative arm were revascularized. Thus, only about 50% of the total population was truly managed entirely conservatively vs. with revascularization. The title of the trial clearly underlines this assertion: Initial Invasive or Conservative Strategy for Stable Coronary Artery Disease.

The second misconception of the ISCHEMIA trial is that it only included patients with very extensive multivessel disease. By CCTA criteria (≥50% diameter stenosis), approximately half of the population had three-vessel disease and proximal LAD involvement. However, if one considers the invasive CAG rather than the CCTA criteria, which is what is actually used for revascularization decisions, one- or two-vessel disease comprised about 60% of cases and proximal LAD disease was present in 36% of patients. This explains why, in a population where >40% had diabetes, PCI was so predominant (74%) compared to coronary artery bypass grafting (CABG) (26%). Furthermore, we do not know the exact details of PCI, which may have been relevant, given that the number of stents or overlapping stents may affect outcomes, especially in the presence of diabetes [35].

The final, and perhaps most important, misconception of the ISCHEMIA trial is that revascularization did not offer any prognostic benefit. When analysing the primary endpoint event curves, two phases are clearly present. During the first months, the curves clearly favour the conservative approach. But, later on, the curves diverge in the opposite direction, favouring the invasive approach. This is because any MI was the major contributor to the primary and major secondary composite outcomes, since there was no difference in all-cause mortality. An invasive strategy increased peri-procedural MI initially, but was later offset by decreased spontaneous MI, which was reduced by 33% at 5 years, a relative risk-reduction strikingly similar to that observed in the FAME-2 trial [32]. Importantly, peri-procedural MI did not have the same prognostic impact as spontaneous MI. Indeed, a subanalysis from the ISCHEMIA trial itself showed that both spontaneous type 1 MI and non-procedural MI were strongly associated with an increased risk of all-cause death (by more than two-fold) and were significantly reduced in invasive-strategy patients, whereas peri-procedural MI, as a whole, was not [36]. Hence, a prognostic benefit was indeed found with revascularization, which is a notable finding, especially considering the previously mentioned caveats.

Lastly, two further insights from the ISCHEMIA trial are worth discussing. Recently, the ISCHEMIA-EXTENDED [37] results were published. This observational study is the long-term follow up of surviving participants from ISCHEMIA. Data from 7-year follow-up reported a lower risk of CV mortality and higher risk of non-CV mortality with an initial invasive strategy over a median follow-up time of 5.7 years. However, in this phase of the study, only the ascertainment of all-cause death can be certain (conducted via participant contact, medical record review or registry search), since there is no longer a central adjudication of events. Considering that the impact of revascularization consisted in the reduction in spontaneous MI, and that the rates of in-hospital mortality of MI are very low today (~4% [38]), it is no surprise that no differences in all-cause mortality were found in the ISCHEMIA trial. Another important finding was the prespecified inclusion of patients with advanced kidney disease [39] where no differences between arms were found for any major endpoints, thereby suggesting that there is indeed little, if any, prognostic impact of revascularization in this subset of patients.

In summary, the aggregate of currently available evidence suggests that there is indeed a prognostic benefit from revascularization in CCS patients, provided there is significant ischemia, as demonstrated by either imaging [5] or coronary physiology [31,32]. The reduction in spontaneous MI has been particularly consistent across trials which have truly fulfilled this prerogative, and it is of both prognostic and clinical significance.

Thus, the commonly defended approach of a routinely conservative approach denies patients this potential prognostic benefit. By contrast, a routinely invasive approach, with demonstration of significant ischemia, enables the assessment of whether a prognostic benefit may be achievable.

Hence, our view of currently available evidence, especially after the ISCHEMIA trial, is not that a routine invasive approach does not offer prognostic value and should not be sought in general, but rather that very good results can be achieved with a conservative approach alone. As a result, should there be clinical (such as reduced life expectancy or comorbidities such as advanced kidney disease) or technical (such as a high risk of complications, low probability of successful revascularization, extensive disease ill-suited to PCI, poor graft landing zone for CABG, among others) factors which might render revascularization problematic, a conservative approach is still very likely to achieve a very good outcome and should be pursued. But, for many patients, if not most, a routinely invasive strategy is more likely to result in the very best of outcomes, when compared to a routinely conservative one, especially on a mid- and long-term basis. To some extent, the current 2023 American guidelines on CCS [6] seem to be in agreement with this perspective, given their class IIa indication for revascularization with the aim of reducing CV events.

A great deal of research is currently underway to identify which subgroups of patients with ischemia are more likely to derive benefit from PCI, such as those with focal vs. diffuse disease [40], or those with vulnerable plaques [41]. Whether these strategies will enable us to fine-tune revascularization or not, only time will tell. But it seems increasingly clear that a personalized approach is indeed important.

#### 2.1.2. Patients with Reduced Left Ventricular Ejection Fraction

Two major trials have addressed the issue of revascularization in patients with reduced LVEF, one with CABG, the other with PCI.

The Surgical Treatment for Ischemic Heart Failure (STICH) trial [42] enrolled a total of 1212 patients with an LVEF of 35% or less and CAD amenable to CABG. The median LVEF was 27%, where about 74% of patients had two or three significant vessel diseases and 68% had ≥75% stenosis of proximal LAD. Initially, there was no significant difference between medical therapy alone and medical therapy plus CABG with respect to the primary end-point of death from any cause. Notwithstanding, in long-term follow-up, (STICHES study—10 years), a significant improvement in all-cause term mortality (10 years follow-up) compared with medical therapy alone (hazard ratio 0.84; 95% CI 0.73 to 0.97; *p* = 0.02) ensued [43]. This difference was further driven by reduced CV events, namely acute spontaneous MI reduction.

An important issue in the STICH/STICHES trial was viability. Its use was mostly undertaken by nuclear exams, occurring in approximately half of the population, but was not mandatory. There were no difference in outcomes in patients with or without viability. LVEF was only mildly improved in patients with viability, with a minimal difference of 2% in absolute terms between the CABG and conservative groups, and with no impact on prognosis. Thus, the findings of the trial suggest that the benefit of CABG came mostly from the reduction in coronary events rather than heart failure events, and that such a benefit seems to be independent from viability.

A relevant limitation to the STICH/STICHES trial, from today’s perspective, was the usage of guideline-directed medical therapy: while the usage of beta-blockers and ACE/ARB inhibitors was high (approximately 90%), no information regarding dosages of MRA inhibition was available. Furthermore, the trial predates ARNI and SGLT2 inhibitors, leading some to raise the question of whether the trial would remain positive today. However, given the above-mentioned findings, and the fact that, from a pathophysiology standpoint, CABG and HF medications seem to work in different pathways, one might argue that the findings of the trial remain very relevant today.

The Percutaneous Revascularization for Ischemic Left Ventricular Dysfunction (REVIVED-BCIS2) study [44] was designed in the aftermath of the STITCH trial, prior to the publication of STICHES. The authors hypothesized that the failure of STICH was driven by two-factors: the burdensome phase of peri-operative CABG, which adversely impacted outcomes in the early stages of the trial; the lack of viability-based revascularization led to increased complexity in the revascularization of non-viable territories. The hypothesis was that PCI could improve event-free survival in patients with severe ischemic left ventricular systole without the early hazard associated with CABG, while viability would appropriately enable the exclusion of territories where revascularization would not be useful, further streamlining the procedure [45].

A total of 700 patients with an LVEF of 35% or less, extensive CAD (defined as a British Cardiovascular Intervention Society [BCIS] jeopardy score of ≥6) and viability in ≥4 dysfunctional myocardial segments were randomized to PCI or conservative management alone. The mean LVEF was 27%, with a surprisingly low percentage of three-vessel disease (40%) and proximal LAD disease (60%). The trial was negative, as the primary outcome, all-cause mortality or hospitalization for heart failure, occurred in 37.2% of the PCI-plus-optimal medical therapy group compared with 38.0% of the optimal medical therapy-alone group (*p* = 0.96). Nevertheless, spontaneous MI was nearly halved in the PCI group (49% vs. 87%), despite the fact that no data were provided regarding the statistical significance of this finding. There were no significant differences in any individual endpoints, including a reduction in ventricular arrythmias. Viability was evaluated mainly by cardiac magnetic resonance imaging (MRI) and clinical outcomes were also the same among those with or without viable myocardium. The extent of viable myocardium was not associated with the primary outcome or any secondary outcome, such as LVEF at 6 or 12 moths and quality-of life-scores. In line with previous evidence [46], scar burden predicted a lower likelihood of left ventricular function recovery and worse prognosis [47].

The REVIVED trial also has a number of caveats worth noting. First, one might argue that the very concept behind the trial was somewhat proven wrong by the STICHES trial, which was published while the REVIVED trial was well underway. Indeed, in the long run, a strategy of complete revascularization regardless of viability had been successful with CABG after all.

Second, many have wondered which patients were eligible for the trial. At its time, extensive coronary artery disease, reduced LVEF and diabetes (approximately 40% of the population) were already known factors favouring CABG [28,48]. Hence, one might argue that the included patients either had less extensive disease (i.e., bystander CAD) or were poor candidates for CABG for either technical or clinical reasons, leaving fundamentally patients who were either “too healthy or too sick” to benefit from PCI. Indeed, the BCIS-Jeopardy Score does enable a patient with a proximal right coronary artery and posterior descending artery lesions to be enrolled, illustrating the concept of “too little” CAD. On the other hand, the authors have publicly shown several cases of extreme CAD, such as patients with three chronic total occlusions, where attempting PCI would be quite debatable.

Third, strictly adhering to viability likely resulted in reduced, non-complete, revascularization. This is highlighted by the reduced number of stents implanted (approximately 330 stents in 330 patients, despite the presence of two- or three-vessel disease in almost 90% of patients), as well as the fact that the reduction in the Jeopardy Score was 71%, thereby theoretically leaving 29% of the myocardium in jeopardy; indeed, there was a trend for improved outcome in patients with a revascularization index of ≥80%.

Lastly, the sample size of the REVIVED trial was somewhat small, especially compared with medical trials or the STICHES trial, thereby hampering its statistical power in assessing differences among groups.

Finally, a finding from the ISCHEMIA trial may also be pertinent. In the subgroup of patients with left ventricular dysfunction (35 to 45% or prior heart failure), an invasive versus conservative approach was associated with a lower rate of the primary endpoint (17.2 vs. 29.3%). Considering that approximately three-quarters of the population underwent PCI and the remainder CABG, this finding emphasizes that the benefit of PCI in patients with abnormal LVEF remains far from settled. Importantly, revascularization was ischemia- rather than viability-based, as the aggregate findings of the study suggest.

In summary, the prognostic benefit of revascularization in patients with heart failure with reduced ejection fraction (HFrEF) remains under debate. While many have pointed out that optimal medical therapy for HFrEF (the so called 4 pillars—BB, ACE/ARNI, MRA, and SLGT2i) has evolved a great deal since the publication of the STICHES trial, the effect of CABG seems to far exceed that of improving systolic function, especially given its impact in the reduction in future MI. Furthermore, the fact that the prognostic effect was independent of viability or ejection fraction improvements further strengthens this notion. Thus, both therapeutic modalities seem complementary, rather than competitive, and the benefit of CABG seems likely to persist today.

As for the role of PCI in patients with HFrEF, further evidence is necessary. Even considering all the limitations of the REVIVED trial, as previously pointed out, it seems rather clear that a strategy of percutaneous revascularization primarily based on viability has failed and should thus not be routinely sought. On the other hand, the seemingly positive findings of the ISCHEMIA trial in patients with prior heart failure or LVEF between 35 and 45%, where revascularization was mostly undertaken by PCI and driven by ischemia rather than viability, provided hope that some patients may indeed benefit from PCI despite an abnormal LVEF.

Only newer trials can test whether a strategy of complete percutaneous revascularization—a true STICHES-like trial, but for PCI—is of prognostic benefit. However, in current clinical practice, a binary approach of CABG only/PCI only is often not employed, but rather a complementary one (selecting the best technical option, including hybrid revascularization). Additional clinical trials are needed, considering the best revascularization approach, which often involves CABG and PCI. Hence, we believe a randomized trial testing complete revascularization vs. medical therapy, selecting the technique deemed best for each case (CABG only, PCI only or hybrid), and not primarily relying on viability for revascularization decisions but perhaps including ischemia testing as an aid, would probably be the next logical step in the field.

#### 2.1.3. Patients with Vulnerable Plaques

A different approach to revascularization would be to treat plaques highly likely to result in acute coronary syndromes, rather than flow-limiting lesions—the so-called vulnerable plaques.

A very recent trial—The Preventive Coronary Intervention on Stenosis With Functionally Insignificant Vulnerable Plaque (PREVENT) [14]—was designed to assess the effect of PCI of non-flow-limiting vulnerable plaques, and greatly merits attention. The authors defined such lesions as those with a percentage diameter stenosis > 50% (by visual estimation) and negative fractional flow reserve (FFR) (≥0.80), plus at least two of the following four intra-coronary imaging characteristics: minimal lumen area ≤ 4.0 mm^2^, plaque burden > 70%, lipid-rich plaque by near-infrared spectroscopy (maxLCBI 4 mm >315) or thin-cap fibroatheroma detected by radiofrequency IVUS or OCT. Patients were randomized to either PCI or medical therapy alone. Both chronic and acute coronary syndrome patients could be enrolled; in the latter, only non-culprit lesions could be selected for randomization. However, given that among the 1606 patients enrolled, 84% presented with chronic coronary syndrome, this trial is essentially applicable to this clinical context.

The primary endpoint (target vessel failure—composite of death from cardiac causes, target vessel MI, ischemia-driven target-vessel revascularization, or hospitalization for unstable or progressive angina) at 2 years was significantly lower in the PCI-plus-optimal medical therapy group: 0.4% vs. 3.4% (hazard ratio 0.11; 95% CI, 0.03–0.36, *p* = 0.0003). There was also a patient-oriented composite outcome (death from any cause, any MI and any repeat revascularization), which was also significantly lower (hazard ratio 0.69; 95% CI 0.50–0.95) in the PCI group. These benefits persisted throughout a 7-year follow-up period. Notwithstanding, while every component of each endpoint occurred less frequently in the PCI arm, the difference only reached statistical significance for ischemia-driven target-vessel revascularization and hospitalization due to unstable or progressive angina.

Several important limitations are worth mentioning. First, of the four imaging criteria which defined a vulnerable plaque, only a plaque burden > 70% and minimal lumen area ≤ 4.0 mm^2^ were present in the vast majority of the population and only 1% of the population fulfilled all four criteria. Secondly, patients underwent IVUS much more frequently than OCT, which may limit the assessment of “true” vulnerability. And third, PCI was performed in 91% of patients assigned to the PCI group, with bioresorbable vascular scaffolds implanted in 33% of patients, which might have partially had a negative impact, given their association with worse outcomes, particularly late stent thrombosis [49]. Thus, the PREVENT trial, while groundbreaking in concept, raises many questions and will require confirmatory trials before the routine PCI of such plaques can be recommended. Notwithstanding, it highlights that flow assessment alone is probably not be enough in ruling out lesions which do not require revascularization. Should other trials confirm its findings, the default rationale may be to revascularize lesions that are either flow-limiting and/or vulnerable, rather than simply flow-limiting or not.

It may also be worth reflecting on indirect evidence from acute MI trials, where the impact of revascularizing of non-culprit lesions is significant. While these are not chronic coronary syndrome patients, non-culprit lesions are not directly involved in the pathophysiology of acute myocardial infarctions, especially in the case of ST-elevation MI (STEMI). Thus, the revascularization of such lesions may be construed as an extreme form of chronic coronary lesion revascularization, perhaps comprising the subset of patients at the highest risk of subsequent spontaneous MI. The fact that approximately 20% of patients in the ISCHEMIA trial had had a previous MI further emphasizes this concept. Furthermore, coronary syndromes are a clinical spectrum, where a mostly chronic setting may be interrupted by an acute coronary syndrome event. Therefore, the strictly binary separation of acute and chronic coronary syndromes is somewhat artificial, from a pathophysiological point of view.

Mounting evidence over the last decade has documented improved outcomes with complete revascularization (i.e., culprit plus non-culprit) with PCI in patients with acute MI, driven primarily by reduced rates of recurrent spontaneous MI. In patients with STEMI, the Preventive Angioplasty in Myocardial Infarction (PRAMI) [50], Complete versus Lesion-only Primary PCI Trial (CvLPRIT) [51], Third Danish Study of Optimal Acute Treatment of Patients with ST-Segment Elevation Myocardial Infarction—Primary PCI in Multivessel Disease (DANAMI-3–PRIMULTI) [52], Comparison Between FFR Guided Revascularization Versus Conventional Strategy in Acute STEMI Patients With MVD (COMPARE-ACUTE) [53] and Complete vs. Culprit-only Revascularization to Treat Multivessel Disease After Early PCI for STEMI (COMPLETE) [54] trials were the most significant studies. In a meta-analysis, including 10 RCTs and 1030 patients [55], a 31% relative risk reduction in CV death was observed with a complete revascularization strategy. The benefit of complete revascularization seems also to be applied to older patients (≥75 years) as well as NSTEMI patients, among whom a physiology-guided approach had a lower risk of CV outcomes than those who received culprit-lesion-only PCI, with the greatest difference once again driven by the reduction in spontaneous myocardial infarction [56].

Only a single trial in this setting, the FFR-guidance for Complete Non-culprit Revascularization (FULL-REVASC) trial [57], which has been very recently published, was negative. It tested an FFR-based revascularization vs. culprit-only approach in STEMI or very-high-risk NSTEMI patients. The trial was terminated prematurely, and the rate of FFR-negative lesions was especially high (>50%), which might partially explain its negative findings. Furthermore, the fact that in both other FFR acute coronary syndrome trials (COMPARE-ACUTE and DANAMI-3 PRIMULTI), a reduction in spontaneous MI was observed, but not statistically significant, together with the negative results of the Multivessel PCI Guided by FFR or Angiography for Myocardial Infarction (FLOWER-MI) trial [58] (which tested an FFR vs. angiography-based revascularization of non-culprit lesions of ACS patients), hint that FFR is likely limited in this particular setting. This is likely the result of the inability of FFR to identify non-flow-limiting vulnerable plaques, thereby excessively reducing the revascularization of lesions which might benefit from intervention. This concept is in agreement with that of the PREVENT trial. The ongoing COMPLETE-2 trial is assessing FFR vs. the angiography-based revascularization of non-culprit lesions of ACS patients and will probably provide a definitive answer regarding the role of FFR in this setting.

In summary, the issue of PCI of vulnerable non-acute plaques is not settled. However, when both evidence from the CCS setting and non-culprit lesions of ACS are considered, it seems that this particular subgroup is likely to become the next target of PCI in the future. Further trials are necessary.

## 3. Revascularizing Patients for Symptomatic Relief

Symptomatic relief is a major objective in revascularization. In almost all trials discussed in the previous sections (COURAGE, FAME 2, ISCHEMIA, REVIVED), there was a significant improvement in either angina symptoms and/or angina-related quality of life, especially among more symptomatic patients. Because most trials have focused on PCI, the evidence base is greater for this modality.

Notwithstanding, symptom relief is subjective, and therefore harder to assess. As a result, the only way of assessing the true effect of revascularization is with a sham procedure. The ORBITA trials did just that.

The Objective Randomised Blinded Investigation with optimal medical Therapy of Angioplasty in stable angina (ORBITA) trial [59] sought to compare PCI with placebo in patients with stable angina and evidence of severe single-vessel stenosis, after a 6-week period of medication optimization. Patients were randomized to PCI or a sham procedure. The trial found that among patients with stable angina, PCI did not result in greater improvements in exercise capacity or angina frequency compared to a sham procedure. However, PCI reduced ischemia more effectively, as ascertained by dobutamine stress echocardiography, and patients with a higher echo score at baseline did experience a greater improvement in angina with PCI [60]. Likewise, in patients undergoing cardiopulmonary exercise testing who had an oxygen-pulse plateau had greater ischemia, and they also experienced greater symptomatic relief with PCI [61]. The ORBITA trial is often considered as evidence that PCI only provides a placebo effect, after all. Yet, another way of looking at it is not that PCI does not improve symptoms, but rather that medical therapy can often be effective enough.

Given the concerns raised by the ORBITA trial regarding the impact of symptoms from PCI, a second trial, ORBITA-2 [62], was undertaken. Rather than adding PCI to medical therapy, the authors sought to assess the effect of PCI independently of anti-anginal medication. Patients were once again randomized to PCI vs. a sham-procedure. Importantly, ischemia was unequivocally documented, as evidenced by the almost ubiquitous use of invasive physiology, together with the highly functional significance of the lesions (with an average FFR of 0.60). PCI resulted in a greater improvement in angina and exercise capacity compared with medical therapy. Eliminating the use of guideline-directed medication as a precondition for PCI was instrumental in isolating the PCI treatment effect. Indeed, in the original ORBITA trial, an aggressive anti-anginal treatment protocol likely rendered the total angina burden too insignificant for PCI to have a meaningful impact. Furthermore, the longer follow-up period of ORBITA-2 (12 weeks, as opposed to 6 weeks of original ORBITA), and the fact that the primary endpoint was the angina symptom score (versus treadmill exercise time) were also key design factors.

Thus, currently available evidence supports the use of revascularization for symptom relief. Current guidelines [3,6] are in agreement and provide a class I indication in this setting. The controversy lies in whether revascularization should be offered upfront as an alternative to anti-anginal drugs, or only to those refractory to medical therapy, as guidelines suggest. Given the extensive controversy surrounding the issue of revascularization in CCS, it seems far from settled. Once again, personalization may be key: encountering a single-vessel focal stenosis, much like the one Grüntzig treated in the very first in-human coronary angioplasty [63], is likely an ideal case for PCI, and should probably be offered upfront. On the other hand, for diffuse, extensive disease, medical therapy will be paramount, and the effect of revascularization on symptoms is far more limited.

## 4. Conclusions

**Figure 1:**
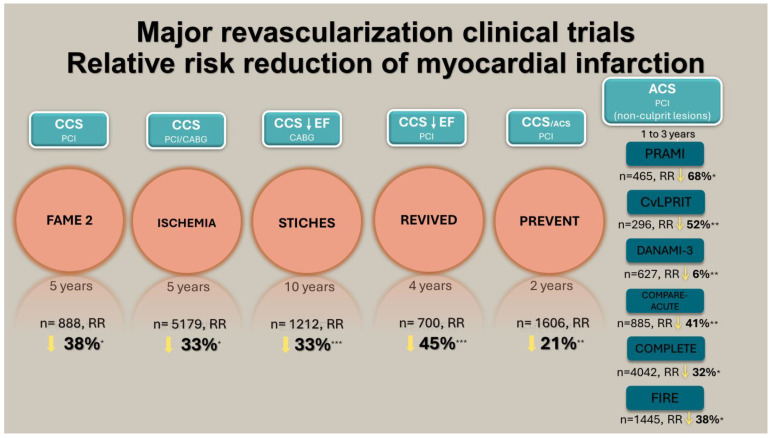
Central Illustration: Relative risk reduction (RRR) for myocardial infarction in coronary artery disease revascularization clinical trials. For the FAME-2, ISCHEMIA, COMPARE-ACUTE and REVIVED trials, the RRR is reported for spontaneous myocardial infarction. For the STICHES, COMPLETE, CVLPRIT, FIRE and PREVENT trials, the RRR is reported for myocardial infarction as a whole. For the DANAMI and PRAMI trials, the RRR is reported for non-fatal myocardial infarction as a whole. * *p*-value and/or 95% confidence intervals reported and statistically significant; ** *p*-value non-significant; *** *p*-value and/or 95% confidence intervals not reported; Abbreviations: ACS, acute coronary syndrome; EF, ejection fraction; CABG, coronary artery bypass graft; CCS, chronic coronary syndrome; PCI, percutaneous coronary intervention; RR, risk reduction.

The issue of revascularization in chronic coronary syndromes remains far from settled. However, we have come a long way since the advent of CAG in the 1950s and 60s [64,65], CABG in the 1960s [66] and PCI in the 1970s [63]. From our point of view, the aggregate of currently available evidence is mostly favourable regarding revascularization in general. First, there is overwhelming evidence supporting the positive impact of revascularization on symptom relief and/or quality of life. Second, the prognostic impact of revascularization has been shown repeatedly, provided there is indeed significant disease, as trials in this setting have consistently shown a reduction in the risk of spontaneous myocardial infarction (Figure 1), which should be a major objective in the treatment of CAD. Very importantly, trials have also shown that medical therapy is indeed very effective for both prognosis and symptoms, and a very good outcome is often achievable with it alone, should revascularization not be deemed appropriate. A great deal of research is still needed, especially for patients with abnormal LVEF, where the optimal approach to revascularization, as well as the exact role of imaging, remains to be defined. Likewise, identifying which patients are more likely to benefit from revascularization and how, with the aid of invasive and non-invasive imaging, will be major factors in the future. Lastly, much greater progress is necessary on the issue of vasospastic and microvascular disease, which very frequently accompany epicardial disease and can be of both prognostic and symptomatic relevance.
